# Influence of Cell Seeding Density and Material Stiffness on Chondrogenesis of Human Stem Cells Within Soft Hydrogels, Without the Use of Exogenous Growth Factors

**DOI:** 10.3390/gels11030213

**Published:** 2025-03-18

**Authors:** Arianna De Mori, Nadide Aydin, Giada Lostia, Alessia Manca, Gordon Blunn, Marta Roldo

**Affiliations:** 1 School of Medicine, Pharmacy and Biomedical Sciences, University of Portsmouth, St. Michael’s Building, White Swan Road, Portsmouth PO1 2DT, UK; arianna.demori@port.ac.uk (A.D.M.); gordon.blunn@port.ac.uk (G.B.); 2 Department of Biomedical Science, University of Sassari, Viale San Pietro 43/B, 07100 Sassari, Italy

**Keywords:** alginate, collagen, hydrogels, chondrogenesis

## Abstract

Mesenchymal stem cells (MSCs) can differentiate into chondrocytes provided with the appropriate environmental cues. In this study, we loaded human adipose-derived stem cells (hAdMSCs) into collagen/alginate hydrogels, which have been shown to induce chondrogenesis in ovine bone marrow stem cells without the use of any exogenous chondrogenic growth factors. We examined the influence of hydrogel stiffness (5.75 and 6.85 kPa) and cell seeding density (1, 2, 4, and 16 × 10^6^ cells/mL) on the chondrogenic induction of hAdMSCs, without exogenous differentiation growth factors. Over time, the behaviour of the hAdMSCs in the scaffolds was investigated by analysing the amount of DNA; their morphology; their cell viability; the expression of chondrogenic genes (RT-qPCR); and the deposition of collagen I, collagen II, and aggrecan. The results showed that all scaffolds supported the acquisition of a rounded morphology and the formation of cell aggregates, which were larger with higher cell seeding densities. Furthermore, the cells were viable within the hydrogels throughout the experiment, indicating that high cell density did not have a detrimental effect on viability. All the conditions supported the upregulation of chondrogenic genes (SOX9, COL2A1, SOX5, and ACAN). By comparison, only the highest cell seeding density (16 × 10^6^ cells/mL) promoted a superior extracellular matrix deposition composed of collagen II and aggrecan with limited production of collagen I. These molecules were deposited in the pericellular space. Furthermore, no histological difference was noted between the two stiffnesses.

## 1. Introduction

Articular cartilage is a specialised viscoelastic connective tissue essential for optimal mobility and function of the joints. It comprises chondrocytes within a composite extracellular matrix of collagens, proteoglycans, and glycoproteins. Its complex structural design, coupled with limited self-repair capability due to avascularity and low cell turnover, make any injury to this tissue a significant contributing factor to the development of degenerative osteoarthritis [1]. Autologous chondrocyte implantation (ACI), one of the first cell-based tissue regeneration therapies to reach the clinic, has been successfully employed to treat cartilage defects; however, its use is hindered by the limited availability of donor tissue, the requirement for in vitro chondrocyte expansion, the need for two consecutive surgical procedures, and the potential subsequent chondrocyte dedifferentiation into a fibroblastic lineage [2,3]. Alternatively, multipotent mesenchymal stem cells (MSCs), sourced from various adult tissues, have garnered increasing interest as promising candidates for cartilage regeneration [4,5]. Nevertheless, the clinical applicability of MSCs for cartilage tissue engineering hinges on our ability to direct their fate towards the desired phenotype before implantation [6]. This approach will reduce the risk of tumorigenic formation from undifferentiated MSCs or fibroblastic differentiation to form fibro-cartilage [7,8]. Traditionally, the in vitro chondrogenic differentiation of MSCs relies on exogenous growth factors, for example, the use of transforming growth factor beta (TGF-β), to induce cellular condensation, akin to embryonic cartilage development [9]. However, challenges persist in achieving a long-term and stable delivery of TGF-β [10,11]. Additionally, the use of TGF-β1 and TGF-β3 may potentially cause cartilage mineralisation, evidenced by the expression of hypertrophic markers (Col X, MMP13, and alkaline phosphatase-ALP) [12]. Studies suggest that cues within the cell microenvironment, such as stiffness, play a role in driving differentiation towards a chondrocytic lineage [11,13]. Mechanical cues can induce cell spreading, changes in cell morphology, the rearrangement of F-actin, stress fibre (actin bundles) formation, adhesion patterns, and differentiation [14]. Hydrogels, offering a protective matrix and biomimetic template, have emerged as promising substrates for the functional remodelling of MSC [9]. It is generally believed that MSC differentiation into chondrocytes occurs when hydrogel stiffness is between 1 and 25 kPa [15]. However, studies investigating the optimal stiffness are inconclusive due to variations in experimental protocols (e.g., static and dynamic culturing environments), making it difficult to draw definitive conclusions for optimal differentiation. For instance, recent studies have shown that when MSCs were grown in static cultures within hydrogels of different stiffnesses, chondrogenic markers were observed to be enhanced at a stiffness of 21 and 25 kPa. However, combining different matrix stiffnesses with cyclic hydrostatic pressure increased the chondrogenic markers in matrices with 17.5 kPa stiffness, with more optimal morphological outcomes seen in softer matrices (5.2 kPa) [15,16,17,18]. Matrix environments with higher stiffness, in the range of articular cartilage (500 kPa), have been reported to better support a chondrogenic phenotype [19]. This indicates the need for further research to determine the stiffness ranges that better support differentiation of different cartilage types. Previously, we developed an alginate-based hydrogel enriched with collagen I that could promote spontaneous chondrogenesis in ovine bone marrow stem cells without exogenous chondrogenic growth factors. The hydrogels were formulated to obtain three different stiffnesses: 0.82, 5.75, and 6.85 kPa. The hydrogels could spontaneously promote the deposition of a cartilage-like tissue without the addition of growth factors, with the highest collagen II and aggrecan deposition observed in 5.75 kPa hydrogels [12]. Our previous study used stem cells from sheep, and further studies are required to investigate the behaviour of human-derived cells in these gels. Human stem cells have been shown to possess different properties compared to cells from other species. For instance, Haddouti et al. compared mesenchymal stem cells derived from ovine and human tissue sources in bone marrow, femoral marrow fat, and adipose tissue. The cells derived from both species showed healthy growth and good immunomodulatory capacity. Positive and negative surface markers were identified to be common for cells from the two different species. Slower osteogenic differentiation was observed in ovine MSCs (oMSCs) compared to human bone marrow-derived MSCs (hBMSCs), whereas chondrogenesis was significantly more pronounced in oMSCs than in hMSCs (from all the cell sources tested) [20]. In this study, we evaluate whether the stiffness of previously developed hydrogels could drive the differentiation of human adipose stem cells down the chondrogenic lineage without the use of exogenous growth factors. Furthermore, we hypothesise that increasing cell seeding densities could support better chondrogenic differentiation of human adipose tissue-derived MSCs (hAdMSCs) within the hydrogels, without the use of chondrogenic factors. Prior research has demonstrated that high cell densities in or on biomaterials confer cartilage-like protein expression (glycosaminoglycans and collagen II), mechanical properties, and cartilage-like morphology of the resulting tissue. For example, using isolated chondrocytes grown on polylactide [21] or in collagen gels [22], the highest cell densities used produce cartilage-like tissue. A high cell seeding density in our soft 3D hydrogel might allow for a close intercellular interaction comparable to that present during embryonic skeletal development, thereby favouring the chondrogenic differentiation of MSCs [8]. The aim of our paper was to investigate how variations in stiffness and cell seeding density influence cell viability, morphology, ECM deposition, and gene expression of hAdMSCs within alginate-collagen I hydrogels in the absence of a chondrogenic medium. To the best of our knowledge, this is the first study examining how both stiffness and cell seeding density influence chondrogenesis in human stem cells within soft hydrogels, without the use of exogenous chondrogenic factors.

## 2. Results and Discussion

### 2.1. Cell Characterisation and Differentiation

Human adipose-derived stem cells presented a fibroblast-like shape and adhered to the tissue plastic, where the average dimensions of the cells were 185.8 ± 85.5 µM (length) with an aspect ratio of 8.69 ± 5.67 (length–width ratio) and a doubling time of 22 h.

**Figure 1:**
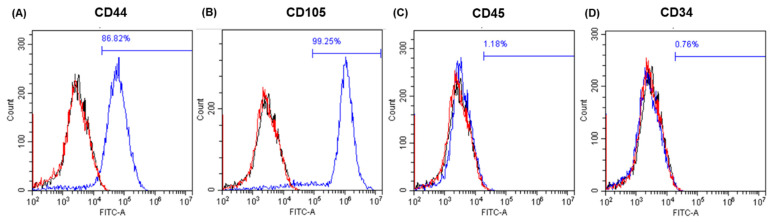
Phenotypic characterisation of hAdMSCs by flow cytometry. The red histograms represent the positive cells out of the total cells analysed (blue line). Negative isotype controls are marked in black. Human AdMSCs were positive for (**A**) CD44 and for (**B**) CD105 and negative for (**C**) CD45 and for (**D**) CD34, respectively.

**Figure 2:**
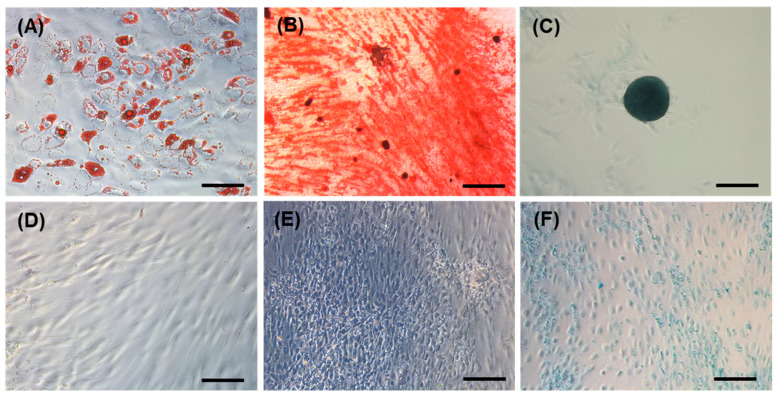
Staining for adipogenesis, osteogenesis, and chondrogenesis of hAdMSCs on day 21. Oil red O staining of cells cultured in adipogenic (**A**) and basal media (**D**). Alizarin red staining of cells cultured in osteogenic (**B**) and basal media (**E**). Alcian blue staining of cells cultured in chondrogenic (**C**) and basal media (**F**). Scale bar is 250 μm.

Immunophenotyping of the cells (Figure 1) was carried out at passage 3, revealing cells positive for CD44 (86.82%) and CD105 (99.25%) and negative for CD45 (1.18%) and CD34 (0.76%). Human adipose-derived cells became adipocytes (as highlighted by lipid droplets stained red with oil red O) in StemPro adipogenic medium (Figure 2A), and none of the control cells, grown in a basal medium, showed any signs of spontaneous adipogenic differentiation (Figure 2D). hAdMSCs successfully differentiated towards an osteogenic lineage using the StemPro^TM^ osteogenesis differentiation kit, as highlighted by its mineral deposits (stained red with alizarin red) (Figure 2B); again, no spontaneous osteogenesis was recorded in the controls (Figure 2E). Chondrogenesis was tested using Alcian blue staining. Figure 2C shows an intense blue staining of the stem cells treated with chondrogenic media compared to their controls in the basal medium (Figure 2F). The controls showed slight blue staining, indicating that low levels of glycosaminoglycans are naturally produced by hAdMSCs. Unlike the cells treated with chondrogenic media, the control cells were unable to spontaneously form pellets.

### 2.2. Evaluation of DNA Content

**Figure 3:**
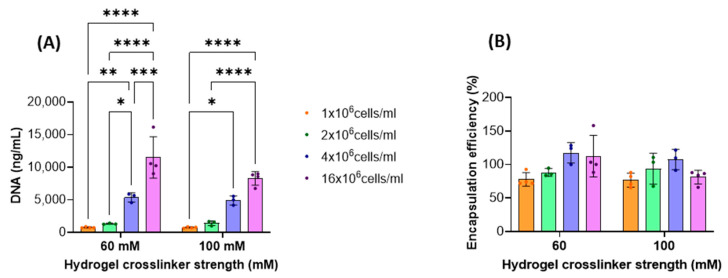
(**A**) DNA content at 24 h after hAdMSC seeding at different cell densities and hydrogel strengths. Data are reported as mean ± SD (*n* = 3). A statistical analysis was carried out between the samples at each cell densities and stiffness by Two-way ANOVA, followed by Sidak’s multiple comparison test (* *p* < 0.05, ** *p* < 0.01, *** *p* < 0.001, and **** *p* < 0.0001). (**B**) Encapsulation efficiency, calculated from the DNA quantity retrieved on day 1 from the gels in comparison to the DNA quantity retrieved from cells cultured in 2D and seeded at the same seeding density. Data are reported as mean ± SD (*n* ≥ 3). Data were analysed by Two-way ANOVA (*p* > 0.05).

**Figure 4:**
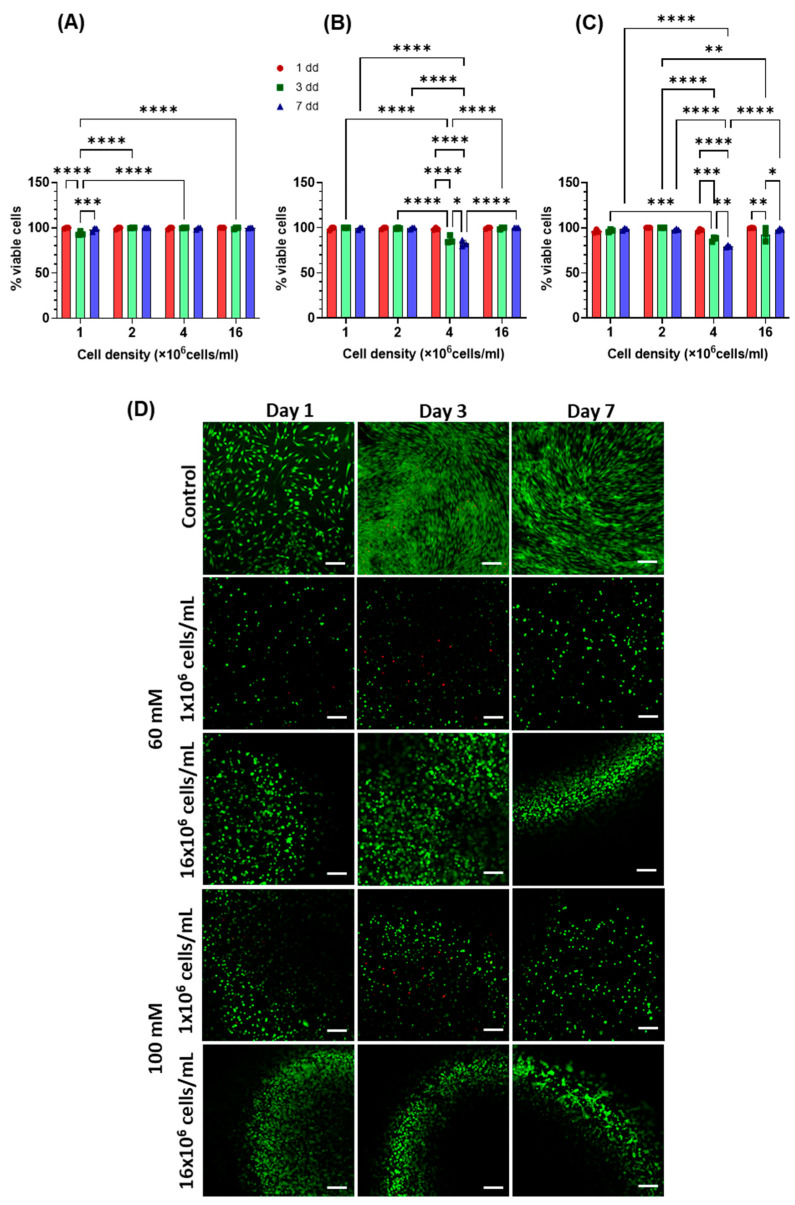
Percentage of viable cells in hydrogels on days 1, 3 and 7 using different seeding densities: (**A**) 2D culture plastic; (**B**) hydrogel crosslinked with 60 mM CaCl_2_; (**C**) hydrogel crosslinked with 100 mM CaCl_2_. Data are reported as mean ± SD (*n* = 3). A statistical analysis was carried out between the samples at each cell densities and stiffness by Two-way ANOVA, followed by Sidak’s multiple comparison test (* *p* < 0.05, ** *p* < 0.01, ***p < 0.001, and **** *p* < 0.0001). (**D**) Representative live and dead images of hAdMSCs seeded at 1 × 10^6^ and 16 × 10^6^ cells/mL density. Scale bar: 265 µm.

Using a 60 mM CaCl_2_ solution as a crosslinker, hydrogels with a stiffness of 5.75 kPa were obtained, whereas employing a 100 mM CaCl_2_ solution, hydrogels with a stiffness of 6.85 kPa were prepared [12]. These gels were loaded with hAdMSCs at four different cell seeding densities: 1, 2, 4, and 16 × 10^6^ cells/mL. DNA quantification was performed to evaluate the proliferation of cells in the hydrogels; however, the loss of cells from the hydrogels due to partial degradation of the scaffold did not allow cell proliferation in the hydrogels to be accurately evaluated (Figure S1). On the other hand, it was observed that a higher number of cells could be seeded and retained within the hydrogels with increasing cell densities (Figure 3A). The encapsulation efficiency was determined 24 h after hydrogel formation (Figure 3B). Cell seeding density and gel stiffness had no effect on cell encapsulation efficiency (*p* > 0.05). Cell viability data confirmed that the decrease in cell number within the gels was not due to the cytotoxicity of the composite material but to the physical loss of cells from the hydrogel (Figure 4). Cell viability was not affected by cell seeding density apart from when 4 × 10^6^ cells/mL were used; at this density, viability was significantly lower in both gels but always higher than 75%, while in all other cases, viability was always above 95%. Cells grown in 2D had a viability higher than 98%. From the live/dead images, gels with a higher cell seeding density tended to form larger cell aggregates, as early as day 1. Furthermore, hydrogels initially seeded with a higher cell number exhibited a greater overall cell population compared to those seeded with fewer cells throughout the duration of the experiment (Figure 4D). This suggests that, despite volume loss due to scaffold degradation, the cell density remained unaltered.

### 2.3. Cell Morphology

**Figure 5:**
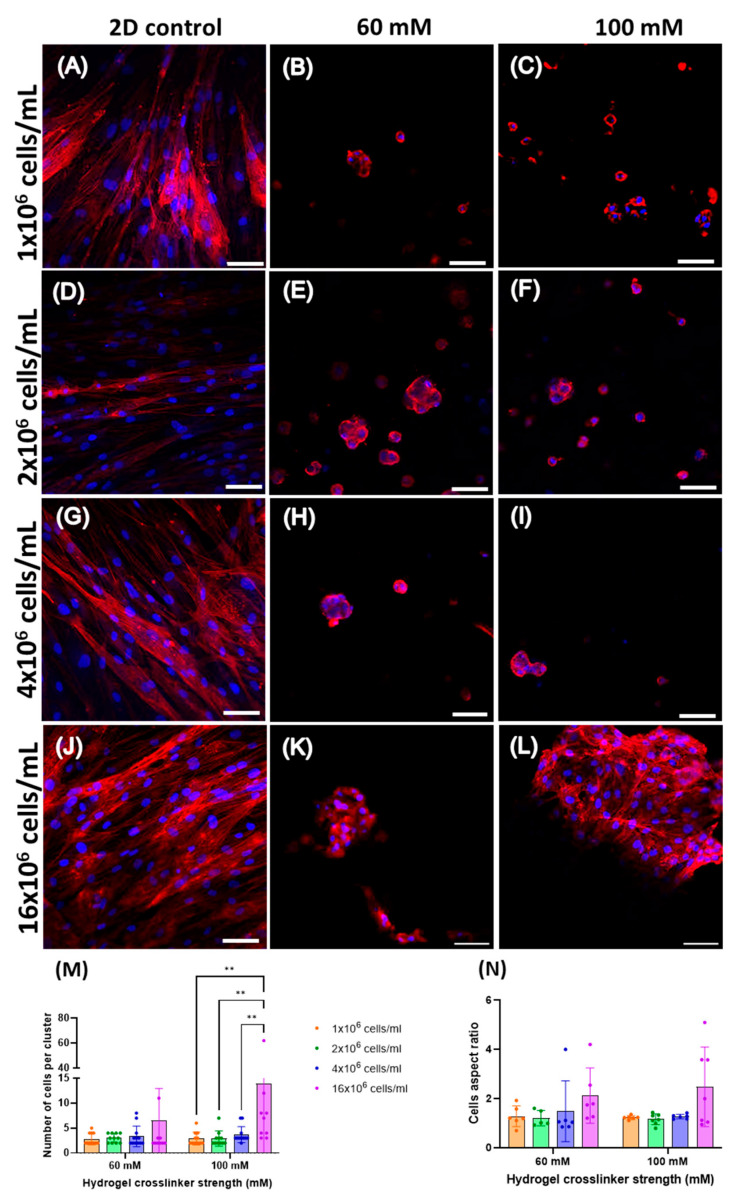
Morphological characterisation (phalloidin and DAPI) of hAdMSCs on day 7 grown in 2D and in hydrogels of two different stiffnesses: 60 mM and 100 mM. The images of hAdMSCs in 2D are shown at increasing cell densities, from to the top to the bottom, in the first column: (**A**) 1 × 10^6^ cells/mL, (**D**) 2 × 10^6^ cells/mL, (**G**) 4 × 10^6^ cells/mL, and (**J**) 16. × 10^6^ cells/mL. Similarly, the cells in gels obtained with 60 mM CaCl_2_ are shown in the second column from top to bottom: (**B**) 1 × 10^6^ cells/mL, (**E**) 2 × 10^6^ cells/mL, (**H**) 4 × 10^6^ cells/mL, and (**K**) 16 × 10^6^ cells/mL. Finally, hAdMSCs, within hydrogels obtained with 100 mM CaCl_2_, are shown in the third column from top to bottom: (**C**) 1 × 10^6^ cells/mL, (**F**) 2 × 10^6^ cells/mL, (**I**) 4 × 10^6^ cells/mL, and (**L**) 16. × 10^6^ cells/mL. Scale bar was 66.25 µm. (**M**) Average number of cells per cluster in hydrogels of two different stiffnesses and four cell densities on day 7. The results are shown as a mean ± SD (*n* ≥ 3). A statistical analysis was carried out between the samples at each cell densities and stiffness by Two-way ANOVA (** *p* < 0.01). (**N**) An aspect ratio analysis comparing cells within hydrogels of different stiffnesses and cell seeding densities. Error bars denote standard deviation, *n* ≥ 3. The comparison between groups was assessed by Two-way ANOVA, followed by Sidak’s multiple comparison test (*p* > 0.05).

**Figure 6:**
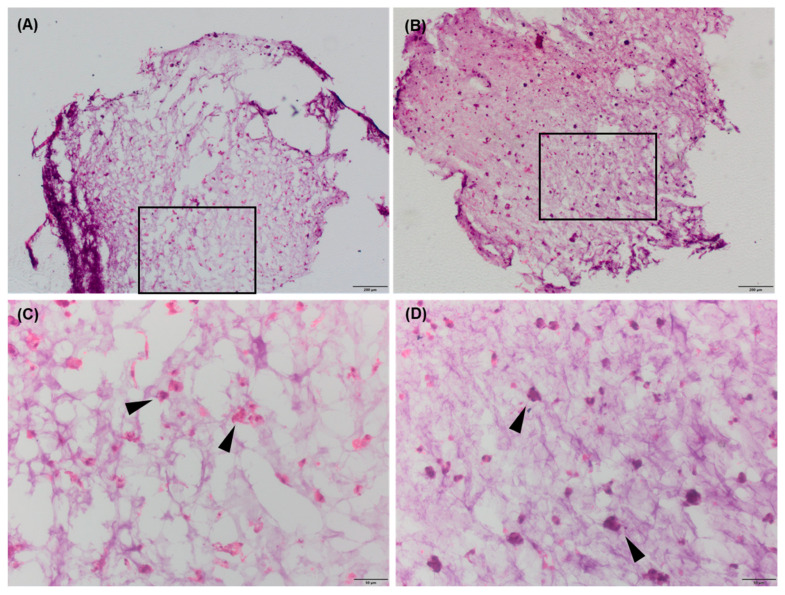
H&E staining of (**A**) 60 and (**B**) 100 mM hydrogels encapsulating hAdMSCs (16 × 10^6^ cells/mL) (scale bar, 200 µm). Purple nuclei were stained with haematoxylin, whereas the cytoplasm and extracellular matrix were stained with eosin. The magnified areas (**C**,**D**) show the presence of cell aggregates (arrows) evenly distributed within the sections (scale bar, 50 µm).

Cell morphology, analysed using confocal microscopy, confirmed the spindle-like shape of human adipose-derived stem cells seeded at different cell densities on tissue plastic (Figure 5A,D,G,J). When in hydrogels, the hAdMSCs acquired a rounded morphology (visible immediately after gelation in CaCl_2_). Although the quantitative analysis found no significant differences in aspect ratio, cellular sphericity was lower within hydrogels with seeding densities equal to or lower than 4 × 10^6^ cells/mL (Figure 5N) on day 7. At a seeding density of 1 × 10^6^ cells/mL, most of the cells remained as single entities in the hydrogels, and very few small aggregates were seen on day 7 (Figure 5B,C). As the cell density increased, larger aggregates (Figure 5E,F,H,I,K,L) formed, even though most of the cells were still individually dispersed, whereas at the highest cell density of 16 × 10^6^ cells/mL, most cells were found in clusters. The number of cells per aggregate increased when the cells were loaded at the higher cell density, with a significant difference for the 100 mM hydrogels with 16 × 10^6^ cells/mL on day 7 (Figure 5M). Moreover, it was noticed that when hAdMSCs were embedded in hydrogels at the highest cell density, the scaffolds better retained their shape compared to hydrogels seeded with lower cell densities. As the scaffolds were mechanically more resistant when incubated with the highest cell density for 28 days, it was possible to section and perform haematoxylin–eosin staining. In cells within the hydrogels of different stiffnesses seeded with 16 × 10^6^ cells/mL, evenly distributed aggregates formed (Figure 6).

### 2.4. Gene Expression

**Figure 7:**
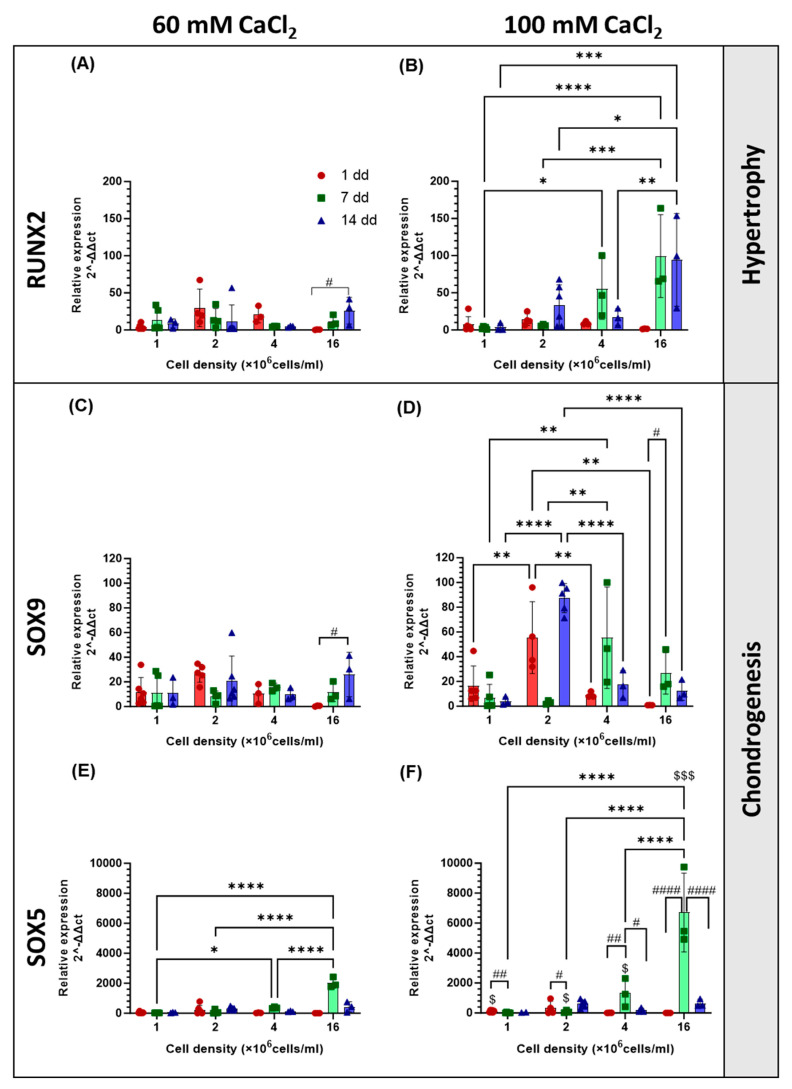
Effect of different cell seeding densities on the gene expression of RUNX2 (**A**,**B**), SOX9 (**C**,**D**), and SOX5 (**E**,**F**) of hAdMSCs within hydrogels of different stiffnesses (crosslinked with either 60 or 100 mM CaCl_2_) on days 1, 7, and 14. Messenger RNA data are presented as fold change expression relative to the 2D controls grown with a basal medium. The results are presented as a mean ± SD (*n* ≥ 3). The comparison among groups was assessed by ordinary Two-way ANOVA, followed by Sidak’s multiple comparison test. * indicated the effect of cell seeding density with * *p* < 0.05, ** *p* < 0.01, *** *p* < 0.001, **** *p* < 0.0001; ^#^ indicates the effect of time with ^#^ *p* < 0.05, ## *p* < 0.001 and #### *p* < 0.0001 ^$^ indicates comparison between the gels of two different stiffnesses with ^$^ *p* < 0.05, ^$$$^ *p* < 0.001.

**Figure 8:**
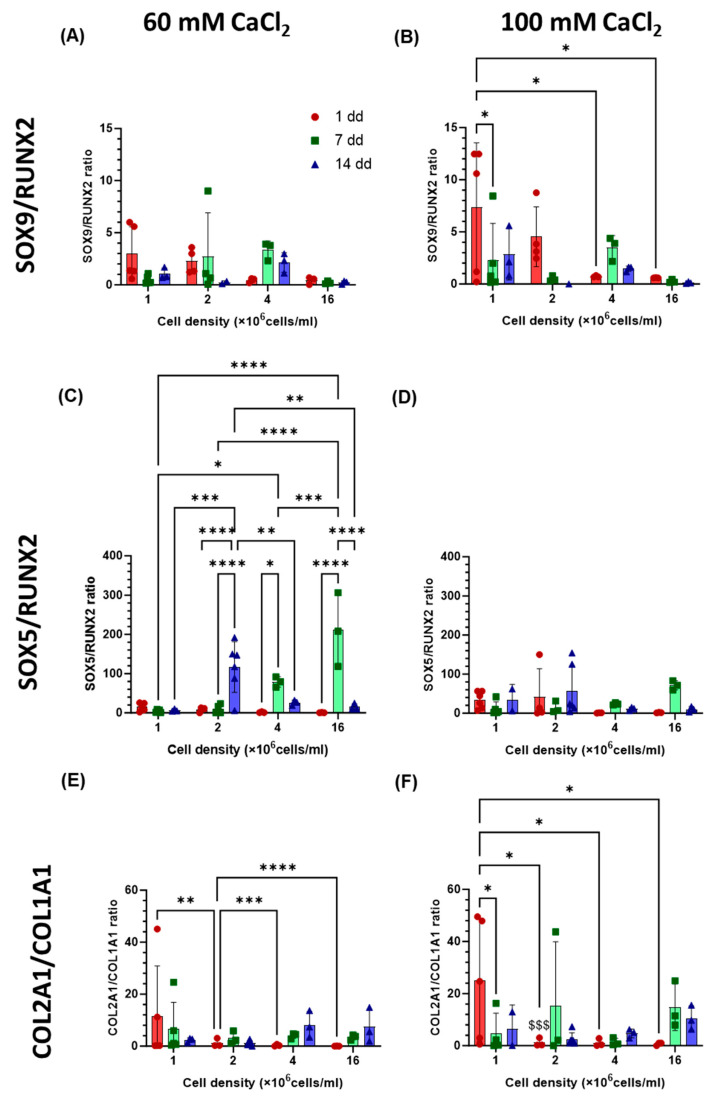
Effect of cell seeding density on the SOX9/RUNX2 (**A**,**B**), SOX5/RUNX2 (**C**,**D**), and COL2A1/COL1A1 (**E**,**F**) ratios. hAdMSCs were seeded on hydrogels with different stiffnesses (60 and 100 mM), and gene expression was analysed on days 1, 7, and 14. The comparison among groups was assessed by ordinary Two-way ANOVA, followed by Sidak’s multiple comparison test. * *p* < 0.05, ** *p* < 0.01, *** *p* < 0.001, **** *p* < 0.0001, $ indicates a comparison between the two gels with ^$$$^ *p* < 0.001.

**Figure 9:**
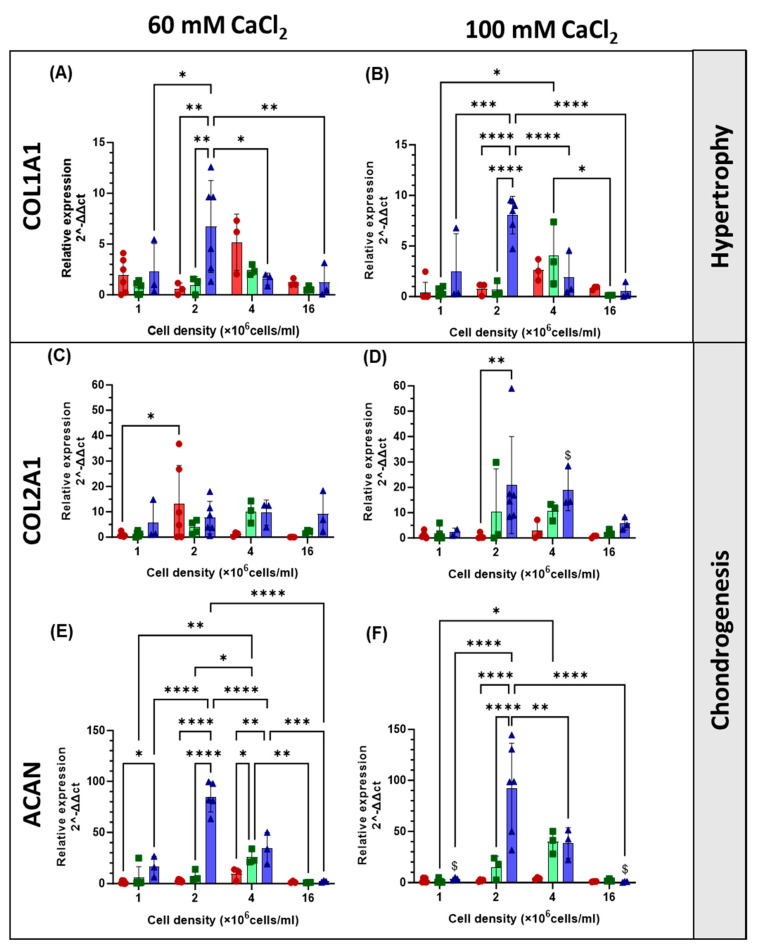
Effect of different cell seeding densities on the gene expression of COL1A1 (**A**,**B**), COL2A1 (**C**,**D**), and ACAN (**E**,**F**) of hAdMSCs within hydrogels of different stiffnesses (crosslinked with either 60 or 100 mM CaCl_2_) on days 1 (red), 7 (green), and 14 (purple). Messenger RNA data are presented as fold change expression relative to the 2D controls grown with a basal medium. The results are presented as a mean ± SD (*n* ≥ 3). The comparison among groups was assessed by ordinary Two-way ANOVA, followed by Sidak’s multiple comparison test. * *p* < 0.05, ** *p* < 0.01, *** *p* < 0.001, **** *p* < 0.0001, $ indicates a comparison between the two gels with ^$^ *p* < 0.05.

The differentiation of hAdMSCs cultured at different cell densities and on scaffolds of different stiffnesses, was investigated by analysing the expression of genes associated with chondrogenesis (COL2A1, SOX9, SOX5, and ACAN) and hypertrophy (COL1A1 and RUNX2) [21]. Compared with cells grown in 2D, SOX9 and SOX5 were both upregulated at all time points and for all the cell densities in the hydrogels (Figure 7C–F). At early time points (day 1 or 7), SOX5 (Figure 7C,D) was significantly overexpressed in the stiffer gel (*p* < 0.05 at 1, 2, and 4 × 10^6^ cells/mL and *p* < 0.001 at 16 × 10^6^ cells/mL) compared to the less resilient one by 140, 217, 354, and 332% for the four increasing cell densities, respectively. Cell density had an effect on the expression of SOX5 in both gels, with increasing expression on day 7 by 25-, 49-, 374-, and 2020-fold in the softer gel and 57-, 183-, 1323-, and 6708-fold in the stiffer gel for the increasing cell densities, respectively. SOX 9 expression (Figure 7E,F) was not affected by the stiffness of the gels (*p* > 0.05). In the softer gel, cell density had no significant effect on the expression of SOX9, while in the stiffer gel, its value was highest (87 fold) at 14 days for cells seeded at a density of 2 × 10^6^ cells/mL, while expression at higher densities peaked at 7 days (55- and 27-fold, respectively). An upregulation of RUNX2 in comparison to the 2D controls in the basal medium (Figure 7A,B) was also observed. Changing cell seeding density did not affect RUNX2 expression in the softer gel; the highest fold increase in expression of RUNX2 was recoded at the highest cell density on day 14 and had a value of 25-fold. In the stiffer gel, the highest overexpression (99- and 94-fold increase) was observed at 7 and 14 days for the highest cell density, with statistically higher expression on day 1, with values of 2-, 6-, 55-, and 99-fold with increasing cell density. In the soft hydrogel, the SOX9/RUNX2 ratio (Figure 8A,B), indicative of the relationship between early chondrogenesis and hypertrophy, was not affected by cell density. Even though there is no statistical difference between the samples, it seemed that the highest cell density favoured hypertrophy. Chondrogenesis was favoured on day 1 for the 1 and 2 × 10^6^ cells/mL seeding densities. The opposite was observed for the SOX5/RUNX2 ratio (Figure 8C,D), indicative of the relationship between late chondrogenesis and hypertrophy; cell density in this case had no effect on the ratio in the stiffer gel, where chondrogenesis was always prevalent. In the softer gel, the highest ratio at 14 days was observed for 2 × 10^6^ cells/mL density, while at 7 days, the ratio was highest for the 4 and 16 × 10^6^ cells/mL seeding densities. The expression of the COL2A1, COL1A1, and ACAN genes, which are responsible for the production of extracellular matrix proteins, was also evaluated (Figure 9). The COL2A1 (Figure 9C,D) gene was always overexpressed on days 7 and 14. A significantly higher expression was observed with stiffer gel on day 14 for the 4 × 10^6^ cells/mL seeding density compared to the softer one. The expression of ACAN (Figure 9E,F) was affected by the stiffness of the gel, with lower expression observed in stiffer gels when either the lowest or highest cell seeding density was used. In both gels, seeding density was a factor in the determination of the expression of ACAN, with the highest expression observed in both gels on day 14 when a seeding density of 2 × 10^6^ cells/mL was used with 85- and 92-fold increases, respectively. COL1A1 (Figure 9A,B) was downregulated, at all time points, for the highest cell density and highest stiffness. At a lower cell density (4.0 × 10^6^ cells/mL), COL1A1 was upregulated at all time points and stiffnesses. The COL2A1/COL1A1 ratio was generally positive across all treatment groups and time points (Figure 8E,F). For both the softer and the stiffer gels, the highest COL2A1/COL1A1 ratio was observed at the lowest cell density (1 × 10^6^ cells/mL) and only at early time points but was not significant on days 7 and 14.

### 2.5. Immunohistochemistry

**Figure 10:**
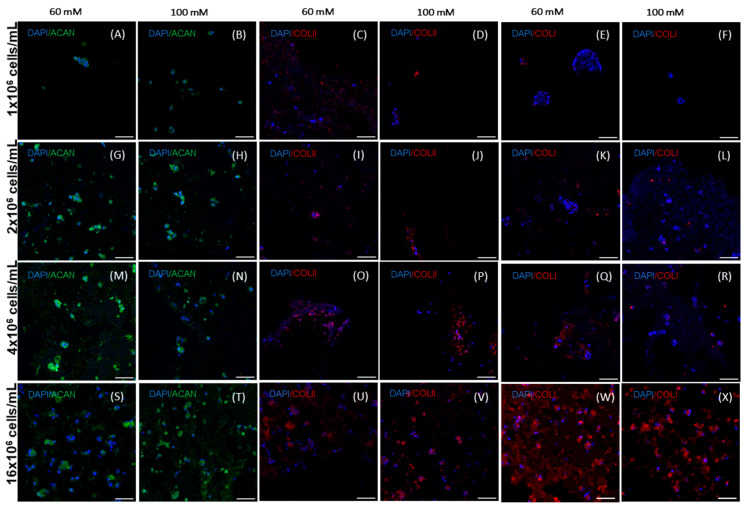
Representative immunohistochemical images of aggrecan (ACAN), collagen type II (COL2), and collagen type I (COL1) of hAdMSCs within hydrogels (60 mM and 100 mM) at different cell densities. The first row shows hAdMSCs cultivated at a cell density equal to 1.0 × 10^6^ cells/mL, the second row equal to 2.0 × 10^6^ cells/mL, the third row equal to 4.0 × 10^6^ cells/mL, and the fourth row equal to 16.0 × 10^6^ cells/mL. Aggrecan staining for the 60 mM hydrogels (**A**,**G**,**M**,**S**) and for the 100 mM ones (**B**,**H**,**N**,**T**). Collagen II staining for the 60 mM hydrogels (**C**,**I**,**O**,**U**) and for the 100 mM ones (**D**,**J**,**P**,**V**). Collagen I staining for the 60 mM hydrogels (**E**,**K**,**Q**,**W**) and for the 100 mM ones (**F**,**L**,**R**,**X**). The scale bar is 66 µm.

Qualitative immunohistochemical analyses were carried out after 28 days of cell culture in the hydrogels (Figure 10). Fewer cells were seen at lower cell densities, and these cells were distributed throughout the hydrogel. However, at higher cell densities, we observed the presence of more and larger cell aggregates. Immunofluorescence staining of collagen type II and aggrecan was used to indicate the production of a cartilaginous extracellular matrix, and collagen type I production, indicating a more hypertrophic differentiation. The results showed that ACAN was present in the pericellular region (Figure 10A,B,G,H,M,N,S,T) across all the cell densities and stiffnesses being tested. Higher cell densities displayed more evident ACAN-stained areas. Collagen type II was scarcely detected at low cell densities, with most of the cells showing no staining. However, at the highest cell seeding density, there was more collagen type II deposition (Figure 10C,D,I,J,O,P,U,V). As with ACAN, collagen type II was mainly present in the pericellular region. In high-density cultures, collagen II and aggrecan were produced around the cells in a pericellular fashion, but at this stage, the formation of a lacunae with a pericellular space, typical of a mature chondrocyte, could not be identified. hAdMSCs within the hydrogels were scarcely positive for collagen type I at low cell seeding densities. Similar to collagen type II, collagen type I production was more evident at the highest cell seeding number (Figure 10E,F,K,L,Q,R,W,X). None of the stiffnesses and cell densities supported homogenous ECM production. No obvious difference in fluorescence intensity was detected between the softer and stiffer hydrogels.

In cartilage tissue engineering, three of the primary challenges are (i) to generate enough chondrocytes or stem cells from limited starting biopsy material, (ii) to either prevent the chondrocytes from dedifferentiating or guide stem cell differentiation specifically towards chondrogenesis, and (iii) to coordinate scaffold degradation with the rate at which the new extracellular matrix forms. In our previous study, we used alginate/collagen hydrogels to support the differentiation of ovine MSCs towards the chondrogenic lineage using three-dimensional scaffolds that could be tuned by modifying their crosslinking and thus their stiffness [12]. The current study used similar 3D hydrogel structures of different stiffness combined with different cell seeding densities of human MSCs.

### 2.6. Three-Dimensional Hydrogel Structures Support Rounded Cell Morphology and the Formation of Cell Pellets

A rounded cellular appearance is closely linked to cell phenotype and is crucial for chondrogenesis, as shown by Zhang et al., who demonstrated that when cytochalasin D (CD) was administered to embryonic stem cells, actin reorganized in stress bundles at the periphery of the cell body and the cells acquired a rounded shape, enhancing the expression of type II collagen, inducing chondrogenesis [22]. Also, de-differentiated chondrocytes, after treatment with CD, re-expressed type II collagen and aggrecan after adopting a spherical shape [23]. In the present work, the human cells encapsulated within the hydrogels consistently displayed a rounded morphology, regardless of hydrogel stiffness or cell density, indicating that the 3D hydrogel environment supports a rounded morphology without the need for specific chemical cues. The second step that is conducive to chondrogenesis is the formation of cell aggregates. A high cell number supports the initial step of the process of chondrogenesis, which involves the aggregation of precursor stem cells, which are tightly bound through adhesion proteins, such as N-cadherin and N-CAM [24,25].

In vitro, the process of cell aggregation can be recapitulated by employing micro-mass culture systems, or pellet cultures; however, these can generate localized heterogeneity and inconsistent chondrogenesis [26,27]. Successful pellets are limited in size, as larger pellets develop a viable periphery but a necrotic centre [28,29]. This could be overcome by merging multiple spheroids to generate larger continuous constructs [30]; however the requirement for a large number of cells represents a significant limitation for clinical application [31]. Alternatively, a cell culture in hydrogel scaffolds can replicate the conditions of high-cell-density pellets, allowing cell contact in the three dimensions [22]; this is desirable as cell density in hBMSC aggregates has been shown to stimulate the expression of glycosaminoglycans (GAGs), early indicators of chondrogenesis, without the addition of exogenous TGF-β1 [32]. On day 7 of our study, the cells were found to have formed aggregates, with larger clusters corresponding to higher cell seeding densities [33,34]. Furthermore, the collagen/alginate gels supported the formation of hAdMSC pellets with no significant difference between gels of different stiffnesses.

### 2.7. Cells of Different Origins Generate ECM at Different Rates

Hydrogels supported the viability of hAdMSCs, with no detrimental effect due to the increase in cell density, suggesting efficient diffusion of nutrients and gases within the volume of the hydrogel, as we previously demonstrated [12,35]. However, the total DNA content within the hydrogels steadily decreased over time, regardless of the initial cell density. Since DNA content decline was not accompanied by an increase in dead cells, this was linked to the migration of cells from the gels, supported by the presence of cells attached to the cell culture plate and within smaller gel fragments. Cell migration was associated with gel degradation due to the diffusion and release of divalent cations in aqueous media, with hydrogel dissolution occurring over time [12,36]. This was also observed for equine and bovine BMSCs in alginate hydrogels crosslinked with CaSO_4_ [37]. However, in our previous investigation, using ovine bone marrow stem cells and the same hydrogels, we observed increased proliferation over time, supported by the deposition of ECM proteins at a rate compatible with the degradation of the gels (80% of gels degraded within 14 days) [12]. Another cause of gel degradation can be the action of enzymes secreted by the cells (i.e., matrix metalloproteinases) [32,38,39,40,41]. For hAdMSCs cultured in collagen/alginate gels, the SOX9, SOX5, ACAN, and COL2A1 genes were upregulated at all time points, with no significant effect caused by cell seeding density or gel stiffness. In regard to ECM production, the expression of aggrecan was generally observed in all samples. In general, independent from the initial cell density, there was deposition of the molecules of interest just in the pericellular region, consistent with previous findings in alginate-based hydrogels containing human MSCs (such as dental pulp MSCs, BMSCs, and Wharton’s jelly MSCs) without growth factors [34,42]. Given the histology and gene expression data, it is likely that low levels of SOX9 and COL2A1 expression might be sufficient to support matrix production in gels with the highest cell seeding density. The gene expression within the hydrogels was overall highly upregulated for the genes involved in chondrogenesis, as previously observed for ovine bone marrow stem cells [12]. However, the inferior deposition of cartilage ECM in hAdMSCs in comparison to oBMSCs [12] poses some questions. It is well known that mRNA presence does not guarantee protein translation because of post-transcriptional regulation synthesis. It is possible, for instance, that the rate of degradation of the scaffolds played an important part in the formation of the ECM. If this degradation happens too quickly, cells do not have the time to form an ECM capable of balancing scaffold degradation. Furthermore, ECM formation could be impaired by the absence of certain ECM components necessary to build proteoglycan aggregates or due to the presence of a high activity of proteases (e.g., MMPs and ADAMTS) that degrade proteoglycans. In our study, hydrogel scaffolds with the highest cell density were mechanically more resilient, indicating that the scaffold was reinforced by ECM production, although the rheology of the scaffolds at 28 days was not measured; this may warrant further investigations in future. Furthermore, despite higher initial cell densities supporting the formation of larger and more numerous aggregates [43], these remained apart from each other, surrounded by the hydrogel matrix, possibly hindering the successive steps of chondrogenesis.

### 2.8. Cells from Different Species Are Sensitive to Different Substrate Stiffness Ranges

Several prior studies have showed how variations in stiffness can differently influence the degree of chondrogenesis in hydrogels, with and without exogenous growth factors. However different authors use different combinations of stiffness values, gel compositions, and cell sources that makes it difficult to draw definitive conclusions. For instance, softer (0.5 kPa) collagen–glycosaminoglycan (CG) (chondroitin sulphate and hyaluronic acid) hydrogels promoted higher upregulation of chondrogenic markers in comparison to stiffer hydrogels (1.5 kPa) using rat mesenchymal stem cells, without the use of differentiation factors [44]. Human adipose-derived stem cell spheroids (81 spheroids of 1000 cells each in 30 µL gel) embedded into methacrylamide-modified gelatin spontaneously deposited glycosaminoglycan after 5 weeks, when the stiffness was between 3.5 and 7 kPa, but not in softer hydrogels. However, in the presence of a chondrogenic medium, superior specific organisation was recorded for 0.5 and 3.5 kPa hydrogels compared to those of 7 kPa stiffness [31]. In hBMSCs (1 × 10^6^ cells/mL) encapsulated into gelatin–hyaluronic acid hybrid hydrogels (crosslinked with H_2_O_2_) of different stiffnesses (ca. 200–600 Pa), the cells were more rounded and produced more aggrecan and SOX9 in the stiffer gels in the absence of a chondrogenic differentiation medium. However, similar to our findings, only the pericellular space was positive for the two markers, and the cells were separated from each other [43]. We chose collagen/alginate gels with 5.75 and 6.85 kPa stiffness based on the success of these gels in guiding the chondrogenesis of ovine stem cells; however, the current study showed that hAdMSCs are not specifically sensitive to this range of stiffness as no statically different results were obtained in terms of gene expression. Several aspects should be adjusted to optimize the observed initial signs of chondrogenesis of these hydrogels without relying on exogenous growth factors. Strategies include mitigating hydrogel degradation (employing diverse crosslinkers and crosslinking densities) [45], exploring higher cell densities, co-culturing MSCs with chondrocytes [46], culturing cells in hydrogels under different degrees of hypoxic conditions [47], mechanical stimulation (dynamic compressive loading and cyclic hydrostatic pressure) [48], and implanting high-density cell aggregates or pre-cultured spheroids within hydrogels [31,49]. Other studies have found that the inclusion of hyaluronic acid or chondroitin sulphate also improved chondrogenesis. Alternatively, Ke et al. found that N-cadherin-modified alginate hydrogels can enhance the chondrogenesis of MSCs via an increase in cell–cell junctions, as well as an inhibition of Wnt-B catenin signalling [24]. Superior cartilage matrix deposition, in the absence of TFG-β, could also be explored by supplementing the media with components that help the chondrogenesis differentiation process, such as dexamethasone (corticosteroid), ascorbic acid (vitamin), and L-proline (amino acid) [50,51].

## 3. Conclusions

This is the first study investigating the impact of both stiffness and cell density on the chondrogenesis of human adipose-derived stem cells within soft hydrogels without relying on the use of exogenous growth factors. Overall, the findings presented support the potential of alginate-collagen I scaffolds in directing encapsulated hAdMSCs toward the chondrogenic lineage. Generally, differentiation appears to be contingent upon cell density, with the highest initial cell number (16 × 10^6^ cells/mL) supporting larger cell aggregates which were positive to ACAN and COL2 after 28 days in culture. However, hydrogels failed to promote the formation of a uniform and intact ECM. Indeed, chondrogenic markers were mainly present in the pericellular space and no cartilage-like organized structure was formed. No difference was observed between the two stiffnesses. Several factors could be explored to try to improve the observed early chondrogenesis of MSCs within the hydrogel without relying on exogenous TGF-β, including the physico-chemical properties of the hydrogels (e.g., types of bonds, degree of crosslinking, the inclusion of N-cadherin motifs, mixing with DECM, and the fabrication of a stratified-design of the constructs), environmental conditions (e.g., changes in the oxygen tension, dynamic stimulation, the dimensions of the hydrogels) and biological factors (e.g., cell seeding number, including its effect on hydrogels stiffness, the use of co-culture with chondrocytes, and the use of Wnt/B-catenin antagonists).

## 4. Materials and Methods

### 4.1. Cell Culture

Human telomerase reverse transcriptase immortalized adipose-derived stem cells (hTERT-AdMSCs) (ATCC^®^ SCRC-4000^™^) were purchased from LGC standards (Teddington, UK). Cells were cultured at 37°C in a humidified incubator (5% CO_2_) in DMEM (high glucose, GlutaMAX™, pyruvate) supplemented with 10% heat-inactivated foetal bovine serum and 1% penicillin-streptomycin (Fisher, Loughborough, UK). The culture medium was replaced twice weekly [52]. At 80–90% confluence, after medium removal, the cells were rinsed with Dulbecco’s phosphate buffered saline without calcium and magnesium (DPBS) (Fisher, Loughborough, UK). The cells were detached following the incubation with trypsin-ethylenediaminetetraacetic acid (EDTA, 0.25%, Fisher, Loughborough, UK) at 37 °C, and then, they were either re-plated (1:2) or stored at −80°C (short term) or in liquid nitrogen (long term). Live cells were counted by trypan blue dye exclusion (Fisher, Loughborough, UK) using a Neubauer improved c-chip disposable haemocytometer (Cambridge Bioscience Ltd., Cambridge, UK). In all sections, ‘controls’ refer to cells that have been grown on tissue culture plastic in DMEM.

### 4.2. Flow Cytometry

Flow cytometry was performed to confirm and identify the surface markers of the hAdMSCs at passage 3. One hundred and fifty thousand cells for each marker were initially rinsed once in the incubation buffer: 0.5% bovine serum albumin (BSA) in cold PBS. Then, they were fixed with 4% paraformaldehyde (PFA) in DPBS (room temperature, 15 min) before being rinsed and stained in the incubation buffer for 1 h. The antibodies used for the staining were CD105-FITC (MHCD10520) (Invitrogen) (Fisher, Loughborough, UK), CD44-FITC (560977) (BD Pharmingen, San Diego, CA, USA), CD34-PE (560941) (BD Pharmingen, San Diego CA, USA), and CD45-FITC (MHC04520) (Invitrogen) (Fisher, Loughborough, UK). The negative isotype controls were MCA928F and MCA929A488 (BioRad, Watford, UK). All the antibodies were used at a dilution of 1:10 in 0.5% BSA in PBS. The cells were then washed three times with PBS and resuspended in 250 µL of PBS. The cells were analysed by CytoflexS (Beckman Coulter Life, Indianapolis, IN, USA), and the data were processed by CytExpert (Beckman Coulter Life, Indianapolis, IN, USA).

### 4.3. Multilineage Potential of hAdMSCs

The differentiation potential of hAdMSCs were investigated *in vitro* for the osteogenic, adipogenic, and chondrogenic lineages. For osteogenic and adipogenic differentiation, 35,000 cells (*n* = 3) were seeded in 24-well plates with DMEM basal media at 37 °C in a humidified incubator under 5% CO_2_. The day after, the medium was substituted with a differentiation medium. For chondrogenesis, 35,000 cells were pelleted in 5 µL of basal media, and the cells were incubated for 4 h at 37 °C in a humidified incubator under 5% CO_2_. Thereafter, the chondrogenic medium was added. For all the treatments, the medium was changed every 3–4 days, and the cells were tested on day 21. Cells that received the DMEM basal media were considered as negative controls. The following media were used for testing the osteogenic, adipogenic, and chondrogenic cell potential: StemPro^TM^ osteogenesis differentiation kit (A1007201), StemPro^TM^ adipogenesis differentiation kit (A1007001), and StemPro^TM^ chondrogenesis differentiation kit (A1007101) (Fisher, Loughborough, UK). For adipogenesis, lipid deposition (21 days) was detected, after fixation, using Oil red O staining (0.3% *w*/*v* in isopropanol) (Merck Life Science, Gillingham, UK). For osteogenesis, mineral deposition (21 days) was determined using alizarin red (34 mM, pH 4.2) (Merck Life Science, Gillingham, UK). For chondrogenesis, proteoglycan deposition (21 days) was detected, after fixation, by staining with 1% Alcian blue in 3% acetic acid (pH 2.5) (Merck Life Science, Gillingham, UK). After washing to remove the staining solution, the cells were imaged using a DMi1 light microscope with an MC170 camera (Leica microsystems Inc., Wetzlar, Germany).

### 4.4. Doubling Time

The cells were seeded onto a 24-well plate (4000 cells per well), and they were allowed to grow overnight. After 24 and 48 h, respectively, a resazurin assay was performed using sterile 0.1 mM resazurin salt solution in a complete medium. Plates were incubated (5% CO_2_ and 37 °C) for 4 h. The blank was the working solution, incubated in the same conditions. Then, 100 µL aliquots were transferred to a new 96-well plate, and the fluorescence was immediately read using a plate reader (ex/em 540/585 nm) (SpectraMax i3x) (Molecular Devices, San Jose, CA, USA). Calibration curves for both ovine and human bone marrow stem cells were carried out (2000, 4000, 8000, 16,000, and 32,000). At experiment completion, resazurin was removed and replaced with fresh medium.

The doubling time was calculated using Equations (1) and (2):

(1) $$\mu =\frac{\ln(\frac{N_{t}}{N_{0}})}{∆t}\cdot 24h$$

(2) $$t_{d}=\frac{\ln(2)}{\mu }\cdot 24h$$

where μ is the growth rate (1/d), Δt is hours of growth (h), N_0_ is the number of cells seeded, N_t_ is the number of cells harvested, and t_d_ is the doubling time (h).

### 4.5. Hydrogel Formulation and Cell Encapsulation

Hydrogels were prepared according to Roncada et al. [12], with a few modifications. Briefly, sterile collagen I from calf skin (Merck Life Science, Gillingham, UK) was dissolved into filter-sterile 2% acetic acid overnight (4 °C) (2% *w*/*v*). Meanwhile, alginic acid sodium salt from brown algae (Merck Life Science, Gillingham, UK) was dissolved into sterile PBS (with 1% *p*/*s*) (5% *w*/*v*) at 4 °C overnight, too. Complete dissolution was achieved by sonication (30–60 min) using a XUBA3 (Grant Instruments, Cambridgeshire, UK). The collagen pH was then brought to 7.4 by the addition of filter sterile NaOH (1M) (Merck Life Science, Gillingham, UK). The pH was monitored with an Accumet AB150 pH metre coupled with a semi-micro pH electrode (Fisher, Cambridge, MA, USA). The pH electrode was sterilised in 70% ethanol (30 min) before use. Finally, the collagen concentration was brought to 0.5% with DMEM. The alginate and collagen solutions were then mixed at a 1:1 ratio, and the cells were added into the viscous solution at different cell densities (1.0 × 10^6^, 2.0 × 10^6^, 4.0 × 10^6^, and 16.0 × 10^6^ per mL). Finally, 50 µL of mixture was added into 250 µL of CaCl_2_ in deionized water (60 and 100 mM) in a low cell attachment 24-well plate. The hydrogels were kept in CaCl_2_ for ten minutes (at room temperature), before removing the excess CaCl_2_, and crosslinking was completed at 37 °C (3 h and 5% CO_2_). Finally, the basal medium was gently added (1 mL per well) and changed every three days.

### 4.6. Live/Dead Staining

Viability of cells was analysed on day 1, 3 and 7 using a phosphate buffered saline solution containing (without Ca^2+^ and Mg^2+^) ethidium homodimer-1 (4 µM) and calcein-AM (2 µM). Briefly, each gel was transferred to a dry and sterile histology slide (previously sterilised with EtOH 70% for 10 min), where a demarking hydrophobic barrier was drawn with a Vector Laboratories ImmEDGE (Vector Laboratories, San Jose, CA, USA) hydrophobic marker pen. Controls on coverslips were kept in the well plate. The staining solution was then added to the gels and controls. The samples were incubated for 1 h at 37 °C, then rinsed twice with PBS (without Ca^2+^ and Mg^2+^), and finally imaged using a confocal laser microscope (LMS 710, Zeiss, Oberkochen, Germany). Gels were kept in PBS during imaging to prevent dehydration. The controls were put onto a slide using Fluoroshield mounting media (Merck Life Science, Gillingham, UK).

### 4.7. DNA Quantification

On days 1, 3 and 7, the hydrogels were moved to new 24-well plates using a sterile spoon. Then, the controls and hydrogels were gently rinsed twice with PBS (without Ca and Mg). The gels were then dissolved with 1 mL of an EDTA (0.1 M) and sodium citrate (0.5 M) sterile saline solution (0.9%, pH 7) at 37 °C (10 min). The samples were then centrifuged at 300 g for 5 min and rinsed, twice, with PBS. The controls in 2D were detached by trypsinisation and rinsed twice with PBS. Thereafter, the cells were lysed using 200 µL of 1% Triton X-100 solution (in 0.2 carbonate buffer, pH 10.2). Complete lysis was achieved by freezing/thawing the solutions (3 times) and passing the extracts through a 21G needle (5 times). DNA was then quantified using a DNA quantification Kit (ab156902) (Abcam, Cambridge, UK), following the manufacturer’s instructions. Briefly, the cell extract solutions were mixed with 100 µL of 1X DNA assay solution in each well of a 96-well plate. The plates were incubated and gently shaken for 2 min at room temperature and protected from light before reading. Bubbles were removed prior to analysis using a sterile needle. Fluorescence was read with a SpectraMax i3x spectrophotometer (480–520 ex/em). The encapsulation efficiency (E.E. %) was determined after 24 h using the following equation:

(3) $$E.E.(\%)=\frac{DNAg}{DNAc}\cdot 100$$

where DNA_C_ is the total DNA in the 2D controls and DNA_g_ is the gels.

### 4.8. Cell Morphology Analysis

We investigated cell morphology by staining the actin cytoskeleton with phalloidin. Briefly, each gel was transferred to a dry and sterile histology slide, prepared as described above. PFA 4% in PBS was then used to fix the cells at room temperature for 30 min. Then, the cells were rinsed and permeabilized with 0.2% Triton X-100 in PBS (with Ca^2+^ and Mg^2+^) for 15 min. Non-specific binding sites were blocked by incubating the cell in 2% BSA in PBS (with Ca^2+^ and Mg^2+^) for 30 min. The cells were stained with Phalloidin Dylight 555 (2 units/mL, in PBS with Ca/Mg) for 1 h. Nuclei were counterstained with DAPI (5 µg/mL) in PBS (with Ca^2+^ and Mg^2+^). Thereafter, the hydrogels were washed twice with PBS (with Ca^2+^ and Mg^2+^). The cells were directly imaged using a confocal laser scanning microscope.

### 4.9. RNA Extraction and Quantitative RT-qPCR

In order to investigate the differentiation of stem cells within the gels, we analysed the expression of genes associated with chondrogenesis (COL2A1, SOX9, SOX5, and ACAN) and hypertrophy (COL1A1 and RUNX2). The cells were lysed using 700 μL of Qiazol (Qiagen, Manchester, UK) and passed through a QIA shredder (Qiagen, Manchester, UK). RNA was then isolated using a RNeasy Plus Micro Kit (Qiagen, Manchester, UK). The total RNA pellet was then dissolved in 22 μL of RNase- and DNase-free water (Invitrogen), quantified using a Nanodrop (Nanodrop ND-1000), and stored at −80 °C until use. A high-capacity cDNA Reverse Transcription Kit (Thermo Fisher, UK) was used to synthesise complementary DNA (cDNA) from total RNA (250–750 ng/μL). The reaction mix and protocol were performed following the manufacturer’s recommendations. RT-qPCR was performed in a LightCycler96 (Roche, Burgess Hill, UK), and the data were analysed using the LightCycler SW 1.1 (Roche, Penzberg, Germany). Each qPCR mixture consisted of Sso advanced Green Supermix (BioRad, Watford, UK), forward and reverse primers, and cDNA template (and water if needed to bring the final volume to 20 μL). The following PCR conditions were used: 95 °C for 10 min, 95 °C for 15 s, and 60 °C for 60 s (45X). A melting curve analysis was performed to determine the specificity of the PCR products. The data were normalised to the expression of the housekeeping gene GAPDH (glyceraldehyde 3-phosphate dehydrogenase). The primers used were GAPDH (housekeeping gene), RUNX2 Runt-related transcription factor 2, COL1A1 (collagen type I alpha 1 chain), COL2A1 (collagen type II alpha 1 chain), SOX5 (SRY-Box Transcription Factor 5), SOX9 (SRY-Box Transcription Factor 9), and ACAN (aggrecan) (Eurofins Genomics, Ebersberg, Germany) (Table 1).

### 4.10. Immunostaining

Protein expression was investigated on day 28 by initially toughening the hydrogels by immersion in 50 mM CaCl_2_ solution for 10 min. After washing once with PBS (without Ca^2+^ and Mg^2+^), the hydrogels with the cells were fixed with 4% PFA for 30 min before rinsing three times with PBS (with Ca^2+^ and Mg^2+^). The hydrogels were then incubated in a 30% *w*/*v* sucrose in PBS (with Ca^2+^ and Mg^2+^) solution at 4 °C for 1 h. The samples were placed in an OCT embedding matrix (CellPath, Newtown, UK), frozen in an isopentane bath previously chilled in liquid nitrogen, and stored at −80 °C. Twenty μm sections were cut with a cryostat (Leica CM3050 S Cryostat, Leica Biosystems, Milton Keynes, UK) and mounted on SuperFrost™ Microscope Slides, left to dry for 30 min, and stored at −80 °C. Staining was preceded by fixation of the slides in ice cold acetone (10 min, −20 °C), followed by 30 min of drying in a fume hood. O.C.T. was removed by gentle rinsing with a 70 mM CaCl_2_ water solution. Antigen retrieval was carried out with the addition of buffer EDTA pH 9 (90–95 °C) for 5 min. Permeabilisation was performed in Triton X-100 0.2% in a 70 mM CaCl_2_ water solution for 10 min. The samples were rinsed once in water with a 70 mM CaCl_2_ water solution and incubated for 60 min in a blocking buffer containing 2.5% *w*/*v* of BSA with a 70 mM CaCl_2_ water solution. The samples were then incubated with primary antibodies overnight at 4 °C in water with 70 mM CaCl_2_. The next day, the samples were rinsed 3 times with a 70 mM CaCl_2_ water solution and incubated with secondary antibodies for 1 h at room temperature in water with 70 mM CaCl_2_. The antibodies were used at the concentrations reported by Roncada et al. [12] and included anti-collagen I, anti-collagen II, and anti-aggrecan. Nuclei counter staining was performed by incubating cells with DAPI (5 μg/mL) for 15 min with a 70 mM CaCl_2_ water solution. The samples were rinsed with PBS (x3) and mounted with aqueous fluorescent mounting media, and pictures were taken with a confocal microscope LSM 710 (Zeiss, Munich, Germany).

### 4.11. Histology

For histology, O.C.T.-embedded sections, obtained as described above, were gently rinsed with deionized water containing 70 mM CaCl_2_ to remove the embedding media. Then, the sections were stained with haematoxylin–eosin (MH51; HT110116; Sigma-Aldrich, Boston, MA, USA).

### 4.12. Statistical Analysis

The statistical analysis details are reported in the captions of the figures. A comparison between groups was assessed by Two-way ANOVA, followed by Sidak’s multiple comparison test, which compares selected pairs of means. The statistical analyses were performed with GraphPad Prism version 10.0.0 (GraphPad Software, Boston, MA, USA, www.graphpad.com, accessed on 12 March 2025). The data were considered significant when *p* < 0.05.

## Figures and Tables

**Table 1:** Primers for RT-qPCR.

	FORWARD	REVERSE
GAPDH	CCGCATCTTCTTTGCGTCG	GCCCAATACGACAAATCCGT
RUNX2	CCACCGAGACACCATGGAG	CGCCTGGGTCTCTTCACTAC
COL1A1	GTTTGGATGGTGCCAAGGGA	AGCACCATCATTTCCACGAG
COL2A1	CCACGCTCAAGTCCCTCAAC	AGTCACCGCTCTTCCACTCG
SOX9	AGGAAGTCGGTGAAGAACGG	CGCCTTGAAGATGGCGTTG
SOX5	AGCAGATGGAGAGGTAGCCA	ACAAGTCTCTTGCGTCAGCA
ACAN	TACACTGGCGAGCACTGTAAC	ACAGGTCCCCTTCGTAGCTG

## Data Availability

The data that support the findings of this study are available from the corresponding author upon reasonable request.

## Supplementary Materials

The following supporting information can be downloaded at https://www.mdpi.com/article/10.3390/gels11030213/s1, Figure S1: DNA quantification in hydrogels loaded with different cell densities: (**a**) 1 × 106, (**b**) 2 × 106, (**c**) 4 × 106, and (**d**) 16 × 106 cells/mL. Data are reported as mean ± SD (*n* ≥ 3). Data were analysed by Two-way ANOVA, followed by Tukey’s multiple comparison test (**, *p* < 0.01, ***, *p* < 0.001, and ****, *p* < 0.0001).
