# Supplementary material for: Influence of Cell Seeding Density and Material Stiffness on Chondrogenesis of Human Stem Cells Within Soft Hydrogels, Without the Use of Exogenous Growth Factors
Source: Gels. 2025 Mar 18;11(3):213. doi: 10.3390/gels11030213 (PMC11941925; doi:10.3390/gels11030213)
Supplement: Supplementary file 1 [file gels-11-00213-s001.zip › gels-3535957-supplementary.pdf]

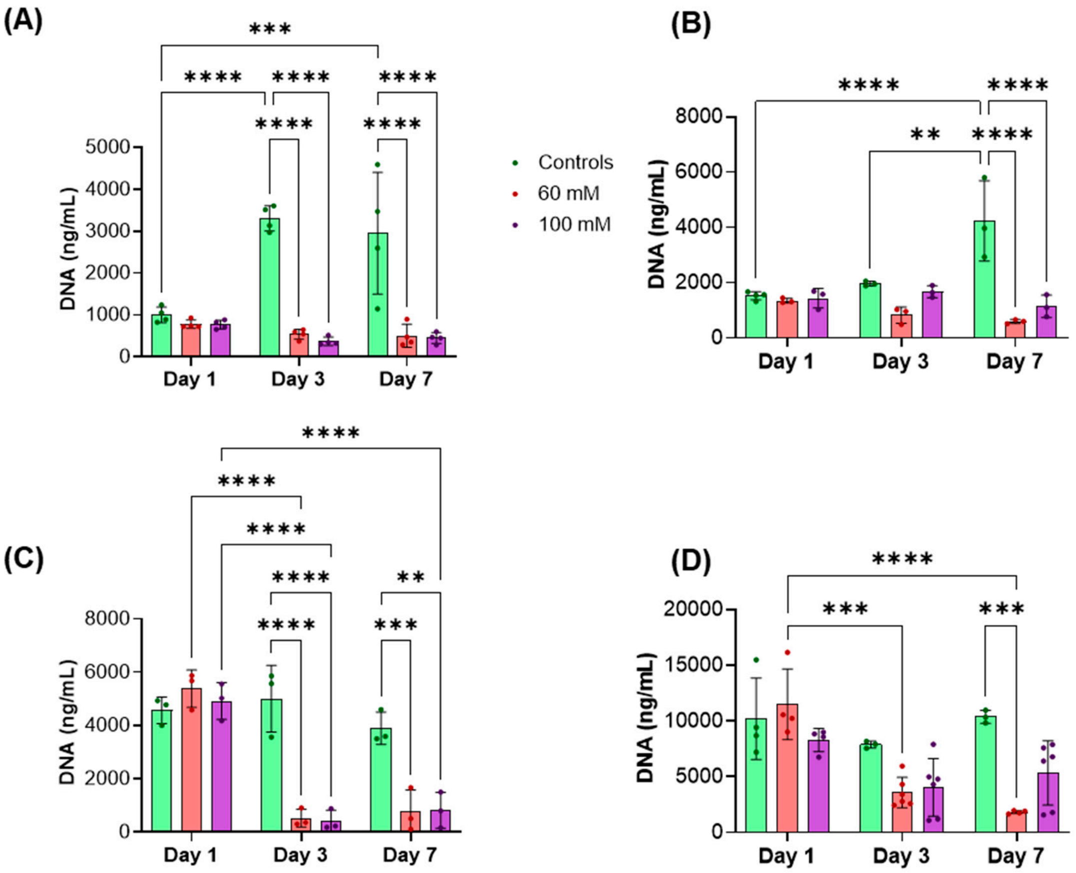

Figure S1. DNA quantification in hydrogels loaded with different cell densities: (a) 1 x 106, (b) 2 x 106, (c) 4 x 106 and (d) 16 x 106 cells/ml. Data are reported as mean ± SD (n ≥ 3). Data were analysed by Two-way Anova followed by Tukey's multicomparisons test (**, p<0.01, ***, p<0.001 and ****, p<0.0001).
